# Why Are No Animal Communication Systems Simple Languages?

**DOI:** 10.3389/fpsyg.2021.602635

**Published:** 2021-03-19

**Authors:** Michael D. Beecher

**Affiliations:** ^1^Department of Psychology, University of Washington, Seattle, WA, United States; ^2^Department of Biology, University of Washington, Seattle, WA, United States

**Keywords:** animal communication, language evolution, animal cognition, animal language studies, information

## Abstract

Individuals of some animal species have been taught simple versions of human language despite their natural communication systems failing to rise to the level of a simple language. How is it, then, that some animals can master a version of language, yet none of them deploy this capacity in their own communication system? I first examine the key design features that are often used to evaluate language-like properties of natural animal communication systems. I then consider one candidate animal system, bird song, because it has several of the key design features or their precursors, including social learning and cultural transmission of their vocal signals. I conclude that although bird song communication is nuanced and complex, and has the acoustic potential for productivity, it is not productive – it cannot be used to say many different things. Finally, I discuss the debate over whether animal communication should be viewed as a cooperative information transmission process, as we typically view human language, or as a competitive process where signaler and receiver vie for control. The debate points to a necessary condition for the evolution of a simple language that has generally been overlooked: the degree of to which the interests of the signaler and receiver align. While strong cognitive and signal production mechanisms are necessary pre-adaptations for a simple language, they are not sufficient. Also necessary is the existence of identical or near-identical interests of signaler and receiver and a socio-ecology that requires high-level cooperation across a range of contexts. In the case of our hominid ancestors, these contexts included hunting, gathering, child care and, perhaps, warfare. I argue that the key condition for the evolution of human language was the extreme interdependency that existed among unrelated individuals in the hunter-gatherer societies of our hominid ancestors. This extreme interdependency produced multiple prosocial adaptations for effective intragroup cooperation, which in partnership with advanced cognitive abilities, set the stage for the evolution of language.

## Introduction

Research programs on animal communication systems in nature have proceeded essentially independently of research programs endeavoring to teach language to animals. This is surprising in light of the early, well-known efforts to relate these two research streams, especially by Hockett (1960) and Marler (1961). These efforts spurred two questions. First, can animals be taught human language, even a simplified version? Second, do the natural communication systems of any animals rise to the level of simple language? Research since then has indicated that these two questions may have different answers: I would suggest a provisional yes to the first, and a provisional no to the second. If this view is correct, it raises a further question: why, then, if some animals can master a version of language, don’t they use this capacity in their natural communication system? In this paper I address this paradox, and make some suggestions toward its resolution.

My paper is divided into four parts. First I consider the main “design features” of language proposed by Hockett as a basis for evaluating language-like properties of animal communication systems. Hockett concluded that some animal communication systems have some of these design features, but none of them have all the design features, especially the key ones. I will designate an animal communication system as a ‘simple language’ system using a variation on the definition of Hewes (1973): “language [is] any system of animal communication which exhibits most of the design features set forth by Hockett” (Hewes, 1973, p. 5). I narrow this definition by identifying four design features – semanticity, arbitrariness, learnability and cultural transmission, and productivity – as necessary for the system to be classified as a simple language. Second, I discuss bird song, a case where several but not all of the key design features are present. I will focus on one specific case of a song-based communication system that is clearly complex and nuanced, but nevertheless lacks three key design features, semanticity, arbitrariness and productivity. Third, I consider the debate, not yet fully concluded, over whether animal communication should be conceived of as a process of information transfer or as manipulation of receiver by the signaler. The debate is germane to our more specific question because it provides a clue as to why we find no simple languages among animals despite the apparent capacity for it in at least some of them. Finally, I suggest that although there appear to be at least some animals with the cognitive capacity for a language-like communication system, none of them have a social system with extreme interdependency among individuals on the scale of that which existed in the hominid hunter-gatherer system. I argue that this extreme interdependency was a necessary condition for the evolution of human language.

## Design Features of Language

In this section I consider the extent to which the most important design features of human language are found in animal communication systems. I use Hockett’s (1960) design features as a basis for comparison of natural animal communication systems with human language. Although Hockett’s design features may have limited use as a theoretical framework for modern evolutionary linguistics (Wacewicz and Żywiczyński, 2015), it is a useful starting point for the comparative analysis of this paper. I have winnowed Hockett’s original design features down to the few I consider the most fundamental ones that can be used to directly compare human language with animal communication systems.

### Specialization: The Purpose of Linguistic Signals Is Communication and Not Some Other Biological Function

Specialization, in Hockett’s sense, is the first defining feature of a communication system, no matter how simple or complex it might be. Otte (1974) defines communication signals as traits “fashioned or maintained by natural selection because they convey information to other organisms”(Otte, 1974, p. 385). I discuss the vigorous debate over the ‘information’ aspect of this definition in Section “Communication: Information or Influence? Mutual Benefit or Manipulation?”, but debaters on both sides would agree that this definition captures the key difference between true communication signals on the one hand, and tactical behaviors or inadvertent cues on the other. For example, while we might describe an individual delivering a blow to a potential opponent as ‘sending a message,’ we mean this only in a metaphorical sense. This behavior is primarily tactical, that is, the individual delivering the blow will directly benefit it if its opponent responds by backing down. If instead of delivering a blow the individual had said “I’m going to kill you,” or growled, or barked, or hissed, we would recognize these as true communication signals, having been shaped by natural selection for the purpose of (literally) sending a message, and requiring adaptations in the receiver as well.

Hockett listed prevarication – the ability to transmit misinformation, i.e., to lie or deceive – as one of his many design features, albeit a minor one, a corollary almost. In Section “Communication: Information or Influence? Mutual Benefit or Manipulation?”, I will argue that we should consider prevarication to be a fundamental, indeed foundational feature of animal communication systems: communication in animals is shaped by the tension between the sender’s and receiver’s interests, and truth in communication is not a given, but rather, when it occurs, hard won.

### Semanticity: Specific Signals Are Directly Tied to Certain Meanings

To say that a communication system is *semantic* is to say that it uses signals to represent particular things or actions. A well-known example in animals are alarm signals given in response to different predators. We can say in such cases that each of these signals represents one of several different predators, or more precisely, the appearance on the scene of one of these predators. For example, vervet monkeys have three different alarm calls for three different classes of predators: raptors, terrestrial mammals and snakes, predators which depend on an element of surprise to capture the monkey. In response to an aerial predator, such as a martial eagle, a monkey emits ‘cough’ calls and sender and receivers take shelter in dense bushes or near the core of a tree. In response to leopards, a monkey emits a ‘bark’ call and the monkeys climb up to the tip of tree branches where leopards cannot safely go. Finally, if a monkey spots a dangerous snake, such as a python, it emits a ‘chutter’ call and the group gathers around the snake, standing upright and harassing it until it leaves the area. Although the vervets use these same signals in other contexts (e.g., intergroup fights) to represent different things, the modification of signal meaning in different contexts occurs in human language as well, and does not negatively impact the representational quality of these signals (Seyfarth et al., 1990; Price et al., 2015). Indeed, it is not unusual for an animal to use a particular signal to mean different things in different contexts (Smith, 1997), similar to some words meaning totally different things within different sentences.

Nevertheless, I will argue later in this paper that the semanticity of animal communication systems is limited: although some things are represented by animal signals, the number of things is generally small. Attempts to catalog the number of different things signaled in animal communication systems typically top out at 25 or so (vervet monkeys, Struhsaker, 1967; Japanese macaques, Green, 1975; review in Hauser, 2000). The limitation does not appear to be due to production constraints (the ability to produce enough distinct signals or to recombine enough of them to enlarge the signal set) or to perceptual-cognitive constraints.

### Arbitrariness: Languages Are Made Up of Arbitrary Symbols Which Have No Intrinsic or Logical Connection to What They Represent

A distinctive feature of human language is that not only are words semantic, they are arbitrarily so. We could equally well call dogs ‘cats’ and cats ‘dogs,’ or any other two words, so long as sender and receiver knew the convention, a point illustrated by the existence of the many different languages of the world. These signals seem totally arbitrary with respect to what they signify, and in theory they could be interchanged without problems, so long as senders and receivers were both aware of the convention. How about animal signals? It appears that in theory we could interchange the vervet alarm signals without problems, provided of course that the receivers were aware of the ‘convention’ (i.e., were hard-wired appropriately). Identity signals – indicating species or individual identity, and occasionally group or kinship – are perhaps the most common animals signals that unequivocally have the arbitrariness feature.

But many, perhaps most, animal signals are not arbitrary. Signals used in agonistic and mate attraction contexts are typically “more of” signals, i.e., more effective signals are louder, longer, bigger, brighter, flashier, designed to impress or to shock and awe. I am unaware of any clear example where the reverse is true, where the more effective signal is the one that is less conspicuous, for example, a softer sound, a more subdued color, a less vigorous display. An apparent exception might be the ‘quiet song’ sung by many songbirds in intense conflict situations, but this typically happens only when the bird is close to its opponent so that the quiet song is audible to the receiver (Searcy et al., 2014); ‘normal’ song is loud because it is a long-distance signal. Moreover, quiet song is typically different in other respects besides loudness, for example, having some elements seen only in quiet song, such as very high frequency elements.

Other animal signals are simple extensions or slight modifications of tactical behaviors, e.g., of attack behavior in agonistic situations. For example, a threat signal in many mammals is the open mouth display, where the teeth, the canines notably, are prominently displayed. Ethologists called this a ‘ritualized’ display (Lorenz, 1966), i.e., one that has been modified by natural selection to be a display, since the mouth is held open, and attack withheld, rather than being the beginning of an actual attack. Another common threat signal is the raising of the hair or feathers, making the animal appear larger. Again, while these actions are plausibly considered ritualized displays, they are not arbitrary signals. If they were, you would also find cases where animals threaten by closing their mouths, or by making themselves appear small. In short, animal signals functioning to impress an opponent or potential mating partner are usually inherently impressive, not arbitrarily selected to represent threat or desirability. Any naïve observer viewing a ritualized dominance interaction between two wolves (or dogs) would have no difficulty determining which animal was dominant and which was subordinate. An upright animal, with its hair raised, its tail raised, and staring at its opponent inherently appears dominant, whereas one with a flattened, slinking body, hair down, tail down, and looking away from the opponent, inherently appears subordinate.

Many epigamic signals – signals designed to attract a mate and induce her to mate – are bright, striking ornaments, often ones that function like supernormal stimuli (e.g., the tail of the long-tailed widowbird, Andersson, 1982). Many epigamic signals are energetically expensive and highly skilled behaviors, such as the complex male courtship dances of wolf spiders and jumping spiders (Hebets and Uetz, 1999; Elias et al., 2012). The motor performance revealed in these sorts of displays likely reflect whole-organism performance relating to survival, and thus should be good indicators of individual signaler quality. There is considerable evidence that females choose mates in nature based upon their evaluations of male motor performance (reviewed in Byers et al., 2010). The relevant point here is that these signals are not arbitrary, but inherently reflect the trait signaled: signaler quality.

Even in the example par excellence of communication of information about the external world – the honeybee dance language – the signals are not quite so arbitrary as generally assumed. For example, if the dance is done outside the hive, where the sun is visible, the bee dances with respect to the actual position of the sun, rather than with respect to the vertical (Gould, 1975). That is, outside the hive, the symbology is not truly arbitrary. Moreover, the distance to the target is represented by the duration of the straight run – the further the distance, the longer the run – so this is at least partially non-arbitrary as well.

Although the words in human language are arbitrary – the existence of different languages is the clearest evidence on this point – they may be expressed in such a way to amplify or otherwise modify their meaning, as for example a loudly shouted “no” indicating stronger conviction. But what would be considered an extra-linguistic feature for humans is often the primary message in animals. For example, the initial stage of a battle between two male red deer consists of a roaring contest (Clutton-Brock and Albon, 1979). This vocal signaling duel does far more than simply establish that each animal is a male conspecific ready to defend or fight for the harem – this undoubtedly was perceived by both parties before the contest began – rather, how loud and how long an individual roars establishes how motivated and formidable he is, and is used by the receiver to decide whether to continue the fight or depart. Similarly, the plumage ornaments and courtship dance of a male golden-collared manakin do far more than simply identify species and sex – that is simply the necessary first step – the brightness of the ornament and the skill of the dance determine whether the receiver, the female, will choose to mate with this particular male or continue her search for the best possible mate (Stein and Uy, 2006; Barske et al., 2011).

In summary, although we have examples of animal signals that are totally arbitrary, many others – perhaps most? – are not. I would add that to date we have found nothing comparable to the many different human languages, which are a consequence of the arbitrariness feature. We do find geographical dialects in animals (e.g., Marler and Tamura, 1964; Wright and Dahlin, 2018), but as the name implies, these are relatively minor variations on the basic signal set, nothing like the wholesale variation seen in human languages.

### Learnability and Cultural Transmission

Human language is both learned and taught. Most animal communication systems are neither. A well-known exception to this generalization are the learned vocal communication signals of several taxa, most notably the oscine passerines (songbirds), hummingbirds and parrots among birds, and cetaceans and at least some bat species among mammals (reviews in Janik, 2014; Knornschild, 2014; Nowicki and Searcy, 2014). Evidence for vocal learning and cultural transmission in some other birds and mammals as well (Walcott et al., 2006; Kroodsma et al., 2013; Stoeger and Manger, 2014; Garland and McGregor, 2020; Barker et al., 2021) suggests that this ability may lie closer to the surface than is generally assumed, but at least at the present time, vocal learning is thought to be rare in animals. Later in this paper I return to the best-studied example of vocal learning, song learning in songbirds.

Where the communication signals are learned, we should expect to find dialects, geographical variation in the signals. The occurrence of dialects is one criterion for identifying the occurrence of learning and potentially evidence for the arbitrariness design feature. An example that may illustrate the arbitrary nature of dialects is the recently-discovered modification of the song in eastern white-throated sparrows to resemble the typical song of western white-throated sparrows. Investigators have traced this change to eastern birds learning the western version of the song on the migration grounds, where individuals of the two populations mix (Otter et al., 2020). Most eastern birds now sing the ‘western’ version of the song on the breeding grounds, illustrating that the details of the song structure are not crucial for its function. Although Otter et al. (2020) suggest that this change might have been driven by a preference on the part of eastern females, they give no evidence for this hypothesis, nor plausible basis for it.

Perhaps even rarer in animal communication systems than learning is teaching. The commonly accepted criteria for demonstrating teaching in non-human animals are that (1) teachers should modify their behavior in the presence of the learner, (2) this change in behavior should result in no immediate benefit to the teacher, and (3) the learner should acquire a behavior quicker or better as a result (Caro and Hauser, 1992). In song-learning studies the birds from whom the young bird learns its song are conventionally referred to as ‘tutors,’ and although live birds are invariably more effective song tutors than recorded song (review in Beecher, 2017), the term ‘tutor’ is used purely as matter of convenience. In fact, in the most common context for song learning in nature, young birds learn from older birds who are or will be their territorial rivals, a very different context from language learning in young humans, where ‘tutors’ are typically relatives or other interested parties who ultimately (but not immediately) benefit from tutoring. Nevertheless, even in the common songbird case where the young bird learns from territorial rivals, bird song tutoring would fit all three criteria for teaching if in fact the older bird reduces his usual aggression when a young bird appears on his territory, increases his counter-singing with the young bird in such a way as to facilitate learning, and benefits down the road from this tutoring (for example, the two cooperate in mutual defense of their territories, or against predators, or refrain from extra-pair mating with one another’s mates). We have indirect evidence for song learning/teaching in song sparrows: mutual survival is greater in young birds and their primary tutor-neighbor (the one from whom they learn most of their songs) the more songs the two of them ultimately share, i.e., the more songs the tutee learned from the tutor, or the tutor taught the tutee (Beecher et al., 2020).

### Productivity: By Combining a Small Number of Meaningless Units Into Larger Meaningful Signals, a Sender Is Capable of Producing Meaningful Statements About Virtually Anything

The sense in which I am using this term is captured by Hauser (2000, p. 448): “the power of [human] language comes from our capacity to take meaningless syllables and combine them into an unbounded number of meaningful words, and then take these words and combine them into an unbounded number of meaningful expressions (Chomsky, 1986; Studdert-Kennedy, 1998).” I will define productivity as recombining a smaller number of basic signal units to produce a larger number of signals, and thus, messages. Indeed, semanticity (representation) and productivity are probably the two central features of human language: by combining basic phonetic units into larger meaningful units, and combining these units further via syntactical rules, we can say almost anything.

Animal communication systems are not productive in this sense, and this is the primary reason we do not refer to them as languages. We would be impressed if a vervet could say something like “Grab your infant and run from the leopard coming from the west but watch out for the python who likes to hide in the bushes just to the east of you.” A human can say this kind of thing easily, combining a relatively small number of atomic units (phonemes) into very large number of basic signals (words) and combining these into a very large set of possible communications. I note that while there is some controversy in phonetics about exactly what are the units of productive combination, there is agreement that all natural languages (including sign language) are made up of meaningless atomic units that are combined into larger meaningful wholes (Zuidema and de Boer, 2009).

Instead of productivity, we could describe the communication system in terms of information capacity. The information capacity of human language is essentially infinite, in the sense that, in theory, we can communicate virtually anything. Our motor, sensory and cognitive capacities obviously will reduce how much information actually gets transmitted and received. But still, the fact is that we can transmit an enormous amount of information with language. Attempts to measure information capacity or information transmission in animals, on the other hand, have given rather modest results. Two estimates of the information about distance and direction in the honeybee dance language have given a high value of 14.9 bits (Gould, 1975) and a low value of 7.4 bits (Schürch and Ratnieks, 2015). My group has estimated the information capacity of the call signature system that parents of the colonial cliff swallow use to find their offspring in their large breeding colonies (Medvin et al., 1993). We estimated the capacity as 8.76 bits, and the estimate would be somewhat larger if we included information that can be derived from visual differences among cliff swallow chicks (Stoddard and Beecher, 1983). The information capacity of human language of course is orders of magnitude larger than this.

We certainly find the potential for productivity in bird song. For example, most songbirds have multiple songs (song ‘repertoires’), and the different songs are made up of different syllables or notes in different orders, and these smaller units can be used in more than one song. Still, although the units are there, and although songbirds may possess the cognitive capacity to comprehend hierarchical structuring in vocal signals (Gentner et al., 2006; but see van Heijningen et al., 2009), they do not use these capacities to form different songs *representing* different things. As Hauser (2000, p. 450) puts it, “in contrast to the recombination of words into sentences by humans, the output of songbird recombination does not change its meaning.” A minor exception are some songbirds who use some song types in a territorial defense context and others in a mate attraction context (e.g., Byers, 1996). As discussed in the next section, theories on the function of song repertoires abound, but they all agree that the different songs function simply to provide diversity, rather than to represent different things.

### Summing Up

Table 1 summarizes the conclusions of this section. The natural communication systems of animals fall short of human language on a number of the key design features of language. They come closest on semanticity, where signals sometimes represent things in the external world or within the signaler, and the signals are sometimes truly arbitrary. However, more commonly animal signals are not arbitrary but inherently meaningful, e.g., an animal making itself appear large is more frightening than an animal making itself appear small. Most animal communication signals and responses are neither learned nor culturally transmitted. And, so far as we know, no animal communication has the *sine qua non* of language: productivity.

**TABLE 1:** Key design features of communication systems (after Hockett, 1960, pruned and combined).

Found in animals?	Design feature	Comment
Yes	Specialization. The purpose of linguistic signals is communication and not some other biological function.	True of animal communication systems, but this is essentially by definition.
Yes but limited	Semanticity. Specific signals are directly tied to certain meanings.	Clear example are the alarm calls given to different classes of predators in a number of species. But the number of different things signaled is typically very small.
Yes but rare	Arbitrariness. There is an arbitrary relationship between a signal and its meaning. There is no inherent relationship between the form of a signal and what it refers to.	Animal signals are sometimes arbitrary. Often they have inherent meaning that can be readily perceived by a naïve observer, e.g., signals used in mate attraction or agonistic encounters that are designed to impress or shock and awe.
Yes but rare	Learnability and Cultural transmission.	Human language is learnable, teachable and culturally transmitted. Bird song appears to be one of the few animal examples that passes at least two of these criteria (teaching still not established).
No	Productivity (based on Arbitrariness, Discreteness and Duality of patterning): language made up of small meaningless units which can be combined into many larger meaningful units which can be combined to say virtually anything.	Some animals appear to have the motor and cognitive capacity for a productive, language-like communication system but they do not use this capacity to develop language-like communication systems.

## Bird Song: Complexity Without Productivity

The oscine passerines (songbirds) are one of the rare animal taxa in which individuals learn their vocal communication signals. In most animals, these vocal signals are ‘hard-wired,’ that is, they develop normally whether or not the animal is exposed to them early in life. It has long been noted that vocal learning in songbirds has many similarities to language learning in humans (Marler, 1970; Doupe and Kuhl, 1999). These similarities include the following. (1) The young bird needs to be exposed to normal species vocal signals in order to produce them as an adult. (2) The sensory phase of song learning precedes the motor phase. (3) Auditory feedback (which can be abolished by deafening) is necessary for the translation of memorized sensory input into motor production. (4) Vocal learning is most efficient in (and sometimes restricted to) a sensitive period early in life. (5) There are specialized parts of the brain dedicated to the vocal control system. (6) Song is socially learned and culturally transmitted, and in at least some cases it may be actively taught (e.g., Carouso-Peck and Goldstein, 2019; Beecher et al., 2020). While notable differences exist among songbird species with regard to the normal progression of song learning (Beecher and Brenowitz, 2005), these six features are essentially true for all of the many songbirds that have been studied to date.

Despite the notable parallels between bird song learning and human language learning, none of the many studies endeavoring to teach a version of human language to animals have focused on songbirds. This is all the more surprising given the language learning shown by Alex the African Gray Parrot, a member of another avian taxon with vocal learning, the psittacines (Pepperberg, 1981, 1987). Moreover, songbirds have strong cognitive capacities, a highly-developed vocal production mechanism, and a vocabulary of basic sound units in their song that rivals or exceeds the basic sound units of human language. There are even songbird species that can mimic human speech sounds (e.g., Hill Mynah birds). On the face of it, all the requisites would seem to be there to support a simple language in a songbird.

### What Is the Function of a Song Repertoire?

In contrast to well-studied white-crowned sparrows and zebra finches, in most songbird species an individual bird will sing multiple songs (has a song ‘repertoire’). For example, song sparrows typically have nine (plus or minus two or so) very different songs. Each of these songs is made up of 5 or 6 distinct elements, and the order of these elements is important (Horning et al., 1993). The songs do not have individual signatures and the nine or so songs in a song sparrow’s repertoire are as different among themselves as would be a collection of songs taken at random one from each of nine or so different birds (Beecher et al., 1994). Song sparrows are somewhere on the middle of the song repertoire complexity scale: many species have larger and even more complex song repertoires. The key point for this discussion is that song repertoires provide clear potential for productivity, as song sparrows and many other songbirds have as many or more distinct units in their vocal communication systems (e.g., about 100 in indigo buntings, Thompson, 1970; and in swamp sparrows, Marler and Pickert, 1984) as there are in human language (a typical language has 40–45 phonemes).

The most popular hypothesis about song repertoires for north temperate zone songbirds – where only males sing – is that they are an epigamic signal produced by males to attract females and that larger repertoires are more attractive than smaller ones (Catchpole, 1987; Searcy and Yasukawa, 1996; MacDougall-Shackleton, 1997; Collins, 2004). Focusing on just the well-studied song sparrow, the evidence for this hypothesis is mixed (Searcy, 1984; Reid et al., 2004; Hill C. E. et al., 2011). The handicap principle, discussed in the next section, would suggest that if large song repertoires are preferred, it is because they are an indicator of some aspect of male quality. Reid et al. (2005) found support for this idea: song repertoire size in male song sparrows correlated with enhanced cell-mediated immune response (CMI) and relative heterozygosity. Anderson et al. (2017) hypothesized that female song sparrows might prefer large-repertoire males because this feature is an indicator the overall learning ability of the male. However, they found no correlations between repertoire size (or two other measures of song learning ability) with an overall measure of learning ability (based on five different learning tasks). I should note, however, that a correlation of vocal learning ability with both overall learning ability and mating success has been found in another songbird, the Satin Bowerbird, a vocal mimic: in this case the vocal learning ability is the ability of males to mimic the calls of other local bird species, both the number of species mimicked, and the accuracy of the mimicry (Coleman et al., 2007; Keagy et al., 2009).

According to another hypothesis, song repertoires play a role in territorial competition, which in north temperate zone songbirds, where only males sing, is largely male-male competition, but outside the north temperate zone where both sexes sing, is pair-pair competition (e.g., Levin, 1996; Langmore, 1998; Logue and Gammon, 2004). There are several hypotheses as to how repertoires might work in the territorial competition context. Song is used by most territorial songbirds at least in part as a keep-out signal, to ‘post’ their territory. Kroodsma (1988) argues that the vocal diversity provided by a repertoire functions to hold the attention of territorial competitors by dishabituating them to the territory owner’s singing, i.e., by holding their attention. As one piece of evidence, he points to a positive correlation between repertoire size and population density in marsh wren populations, and also to the finding that birds in denser populations cycle through their songs faster, again a behavior that should reduce habituation (Kroodsma, 1977). In contrast, song sparrows sing their much smaller repertoires with eventual variety, i.e., singing each one of their song types many times before switching to another type, and this would seem to argue against the dishabituation hypothesis. In western, resident populations of song sparrows, song repertoires may function primarily to provide a bird with songs matching all (or most) of his neighbors, and thus potential individualized replies to each one of them (Beecher et al., 1997; and see next section).

Although as this brief discussion indicates, the theoretical debate has not yet concluded, the take-away point is that none of these hypotheses view song repertoires as a form of semantic communication. Rather they view repertoires as having a direct effect on the receiver (dishabituation), or as permitting individualized replies to multiple neighbors, or as quantitative signals with inherent rather than semantic meaning, that is, more songs (or more song syllables) are simply more effective.

I should add that most single-song species appear to have the potential to develop song repertoires yet do not tap into this potential. For example, when examined over an entire population, indigo buntings have a repertoire of over a 100 distinct song syllables, yet a given individual uses just 6–8 of these in the single song it develops (Rice and Thompson, 1968; Thompson, 1969; Baker and Boylan, 1995).

### An Example: Communication in a Negotiation Context

Although the different songs in a bird’s repertoire do not have different meanings, a bird having a song repertoire can still use the different songs to communicate in more subtle, nuanced ways than might at first be suspected. In this section I describe one such case: how song sparrows use the songs in their song repertoire to negotiate territorial disputes. The general point I will make is that their communication system is surprisingly complex and versatile, despite being neither semantic nor productive. Although I will not attempt to generalize to all songbirds given the incredible diversity of the song communication systems seen in this group (Beecher and Brenowitz, 2005), I suspect that this conclusion – complexity without productivity – applies broadly to songbirds, and perhaps to all animals.

Song sparrows have a territorial system like that found in many animals and typical of many songbirds. An individual carves out a territory where the mated pair will nest and raise their young, doing most of their feeding on the territory. Suitable habitat is typically densely occupied by conspecifics, so territorial disputes can arise during both the establishment and maintenance stages. The relationship between territorial neighbors can become relatively non-hostile once established, however, on the principle that the enemy you know is better than the enemy you don’t know, generally referred to as the ‘Dear Enemy’ relationship (Fisher, 1954; Akçay et al., 2009, 2010; Beecher and Akçay, 2014). Because in territorial animals, neighbors have no fences, neighbors need to renegotiate territory boundaries from time to time. Negotiation can progress into fighting but avoiding fighting may benefit both parties and this common interest favors reliable signaling. Therefore, as I will discuss in Section “Communication: Information or Influence? Mutual Benefit or Manipulation?”, we should expect to find some degree of honest communication concerning not only fighting ability (resource-holding potential) but also motivation to fight (e.g., at a particular point in time, one party may have more to lose than the other).

**FIGURE 1:**
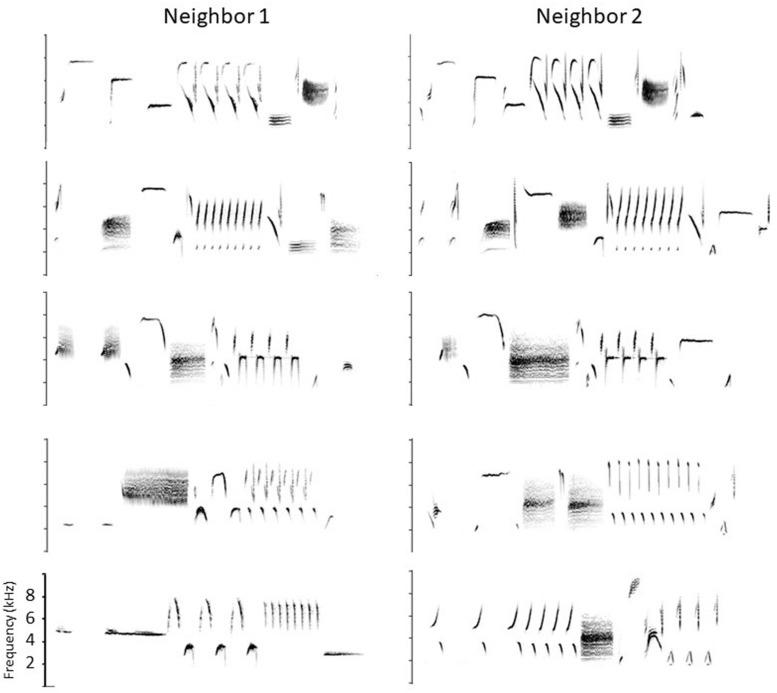
Partial song repertoires of two neighboring birds. Shared songs are shown in the top three rows, and four of their unshared songs in the bottom two rows (they are arbitrarily paired). Frequency scale: 0–10 kHz. Songs are 2–3 s long.

Song sparrows in western, resident populations use their repertoires in a complex way to carry out territory negotiations. Although they will engage in serious fights, established neighbors use their signaling system to avoid fighting if possible. Before fighting they typically give their high-level threat signals, wing waves and soft song (Searcy and Beecher, 2009; Searcy et al., 2014; Akçay et al., 2015a). But before reaching this stage, they use the songs in their repertoires to escalate or de-escalate the dispute following a set of ‘conventions’ predicated on which songs the two birds happen to share (Beecher et al., 1996, 2000; Burt et al., 2001, 2002; Beecher and Campbell, 2005; Akçay et al., 2011; Templeton et al., 2012; Akçay et al., 2013, 2015b). Because western song sparrows learn songs from their neighbors in the area to which they disperse after fledging, a bird typically shares some of his songs with each of his immediate neighbors. The set of songs he shares with one neighbor is typically different from the set he shares with another. A partial example is shown in Figure 1. For example, if we represent the different songs of a bird with different capital letters, and the shared songs of neighbors with the same capital letter, then Bird 1 might share his song types A, B, and C with his neighbor Bird 2, his song types C, D, and E with another neighbor, his song types E and F with a third neighbor, and finally G, H, and I with no neighbors (e.g., the bird he learned these songs from may have died). A typical territorial negotiation might occur as follows. Suppose Bird 1’s mate finds an ideal place to build her nest just over the previously-established boundary with Bird 2. Bird 1, aiming to establish this new boundary, moves to that point and sings at his neighbor. Typically the two birds would still be a considerable distance apart at this point and out of sight of one another (territories are large and song is a long-distance signal). Although Bird 1 could sing any one of his 9 songs to Bird 2, in this circumstance he would typically ‘address’ Bird 2 by singing one of their shared types, A, B, or C. Let us say bird 1 sings B. Bird 2 can escalate by replying with his B’ (i.e., his most similar song to Bird 1’s B). This ‘type match’ is a low-level threat signal and would be the first step in escalation. Alternatively, he could ‘confirm’ without escalating by replying with A’ or C’ (‘repertoire matches’, Beecher et al., 1996). Note that this type of reply is only possible if Bird 2 knows Bird 1 well enough to know which songs they share and which songs they don’t. Finally, rather than type-matching or repertoire-matching, Bird 2 can de-escalate by singing one of his unshared types, e.g., D, E, F, G, H or I. Singing an unshared type is better than not singing at all because it signals that although the singer is not engaging, he is on territory and has heard his neighbor; it is a signal likely used for example when the bird is busy feeding recently-fledged young. If Bird 2 does type match bird 1 (sings B’), Bird 1 in turn can continue to sing that song type (‘stay on type’), or he can de-escalate by switching to another shared song (A or C, ‘repertoire match’), or de-escalate further by switching to an unshared type (e.g., D or E), or disengage totally by stopping singing.

Each ‘convention’ – type matching, repertoire matching, staying on type, switching to an unshared type – has a distinct signaling function in this graded signaling system, with both type matching and staying on type when type-matched signaling a readiness to escalate, repertoire matching signaling recognition of the sender and engagement but stopping short of escalation, and switching to an unshared type signaling de-escalation. The system while not in itself resolving anything, does give the neighbors time to defuse the situation or work out a compromise. Note, however, that the semantic content is limited. No particular song in the repertoire means a particular thing. A song’s meaning is defined entirely by the context of who the receiver is, and even then there are essentially only three meanings, roughly ‘back off,’ ‘I hear you and know who you are,’ and ‘I’m busy now.’

### Summing Up

Songbirds check several of the design feature boxes and they would appear to have the potential to use their songs in a productive way, i.e., to use their signaling system to say many things. However, despite considerable debate concerning the function of song repertoires, the different repertoire hypotheses all agree on one point: that the function of the vocal diversity is diversity *per se*, not the transmission of different messages with different songs. Perhaps even more surprising, many single-song species have large song syllable repertoires an individual could tap into, but instead each individual uses just several of these syllables to develop its single song. No songbird rearranges its multiple song syllables into different songs that signal different things. I echo here the conclusion of Fitch and Jarvis (2013, p. 502): although songbirds (and parrots) have vocal learning and a complex vocal repertoire, they do not “use their songs to communicate combinatorial propositional meanings, i.e., semantics.”. Songbirds may use their repertoires in subtle, nuanced ways, as with the song sparrow hierarchical signaling system I described above, but what the system achieves seems better described as the management of behavioral conflict than as an impressive transmission of information. That is, the system may function well, but it does not function like a language.

## Communication: Information or Influence? Mutual Benefit or Manipulation?

In this section I discuss the debate within the field about the fundamental nature of animal communication. I believe this debate has provided us with a key to understanding why we find no examples of a simple language among the many communication systems of non-human animals, and true language only in the human animal.

We can trace the real beginning of the field of animal communication to the classical ethologists (e.g., Tinbergen, 1952). The ethologists provided detailed descriptions of animal signaling systems in nature, developed theories about the underlying proximate causes (e.g., sign stimuli, innate release mechanisms, and fixed action patterns) and evolutionary processes (e.g., ritualization), and most relevant here, established the view of animal communication as – like human language – an information transfer process. On the question of the function of animal signaling systems, they took a group-selectionist perspective: the benefit that a signaling system provided went not to signaler or receiver *per se*, but to the species (see Tinbergen, 1964 definition in Table 2).

**TABLE 2:** Definitions.

Tinbergen, 1964	“One party… emits a signal, while the other party… responds in such a way that the welfare of the species is promoted.”
Marler, 1968	In “true communication… both parties seek to maximize the efficiency of information transfer.”
Otte, 1974, p. 385	Signals: “behavioral, physiological, or morphological characteristics fashioned or maintained by natural selection because they convey information to other organisms”
Dawkins and Krebs, 1978, p. 283	“Communication is said to occur when an animal, the actor, does something which appears to be the result of selection to influence the sense organs of another animal, the reactor, so that the reactor’s behavior changes to the advantage of the actor.”
Green and Marler, 1979, p. 73	“Communication consists of the transmission of information from one animal to another.”
Krebs and Dawkins, 1984, p. 401	They call the sender role the ‘manipulator’ and the receiver role the ‘mind-reader.’ “The manipulator role is selected to alter the behavior of others to its advantage, the mind-reader role to anticipate the future behavior of others.”
Smith, 1997, p. 11	Communication: “any sharing of information between entities—in social animals, between individual animals”
Bradbury and Vehrencamp, 1998, p. 3	True communication: “information exchange from which both sender and receiver benefit.”
Maynard Smith and Harper, 2003, p. 3	A signal is “any act or structure that alters the behavior of other organisms, which evolved because of that effect, and which is effective because the receiver’s response has also evolved.”
Owren et al., 2010, p. 771	Animal Signaling: “the use of specialized, species-typical morphology or behavior to influence the current or future behavior of another individual.”

Following the revolution of the 1960’s and 1970’s first known as sociobiology (Wilson, 1975) and subsequently as behavioral ecology (Krebs and Davies, 1978), natural selection came to be viewed as acting on individuals, rather than species or groups (Williams, 1966). For some researchers, the shift from naïve group selection to individual selection did not entail a significant change in view: it was simply assumed that signaler and receiver both benefited from the transmission of information, and so this basic parallel with human language was maintained (see Table 2 definitions of Marler, 1968; Otte, 1974). The assumption of mutual benefit seemed natural in cases where sender and receiver have a strong common interest, e.g., the honeybee ‘dance language’ where scout and recruit are both working toward the same end, to provide food for their relatives in the hive. But as investigators began considering the many cases where signaler and receiver have conflicting interests, such as in agonistic encounters over an indivisible resource, they began to question the mutual-benefit, information transmission view. They asked two questions about such cases. First, do both parties have to benefit? Second, do we need to even talk about ‘information transmission’? Isn’t the signaler simply selected to manipulate (or influence) the behavior of the receiver to its advantage? The manipulation viewpoint was famously developed by Dawkins and Krebs (1978) who argued that rather than expecting signalers to signal honestly, we should expect them to manipulate the receiver to their own advantage, e.g., to convince opponents to retreat, or potential partners to mate with them.

Since the Dawkins and Krebs (1978) paper, the debate has continued as to whether it is justified or productive to conceptualize animal signaling as an information transmission process in which both parties benefit. Simplifying somewhat, I will distinguish between the Information Transmission and Manipulation approaches to animal communication. Strong arguments on the manipulation side since Dawkins and Krebs (1978) include Krebs and Dawkins (1984), Owings and Morton (1998), Scott-Phillips (2008), Rendall et al. (2009), and Owren et al. (2010). Strong arguments on the information side over this same period include Green and Marler (1979), Smith (1997), Bradbury and Vehrencamp (1998), Searcy and Nowicki (2005), Carazo and Font (2010), Seyfarth et al. (2010), and Wiley (2013). Definitions from some of these sources are included in Table 2.

In conceiving of signaling as manipulation, Dawkins and Krebs (1978) essentially treated the communication interaction like a zero-sum game. This seems reasonable in cases like disputes over an indivisible resource (a food item, a territory, and a mate), and also in epigamic selection, where a male tries to persuade a female to mate with him now rather than to continue searching for a possibly better male. Although the manipulation view was enlightening in many respects, as originally presented it had a serious weakness: it gave no agency to the receiver. While it was sensible to expect signalers to signal for their own benefit, why should we expect receivers to be passive in these evolutionary scenarios, especially if being manipulated by the signaler is costly? Rather, we should expect receivers to show ‘sales resistance’ to signals that carry misinformation or are pure propaganda (“I am the best,” “I will fight you to death”). Indeed, receivers can do more than simply ignore signals that do not benefit them: they can require signals that do benefit them, even if those signals are costly to the sender. For example, in many species males must sing or call to attract a female for mating. If the male does not vocalize, potential female receivers will simply not engage. Moreover, these vocal signals may attract predators, a cost borne by the signaler but not the receiver. Indeed, the most effective or most-preferred signals may be the most costly, e.g., most conspicuous not just to the intended receiver but to predators as well. This is the case for a male túngara frog (Ryan and Rand, 1990). Males attract females to mate with a ‘whine’ call or a ‘whine-chuck’ call. When a male adds chucks to his calls, he not only attracts more females, but also predators: frog-eating bats that home in specifically on the chucks. Similarly, a calling male field cricket attracts more females than does a silent male, but he also attracts more parasitoid flies, and louder calls attract both more females and more parasitoid flies (Cade, 1975). In some populations the rate of fly parasitism is so high that males have lost the ability to sing (Zuk et al., 2006). As another example, territorial animals often vocalize as a “keep-out” signal. When a territorial songbird is deprived of its voice, however, potential rivals show up and proceed to take over its territory (e.g., McDonald, 1989).

If we reframe our view of the communication system as beginning with the implicit requirement that the receiver imposes on the signaler—to signal—rather than with the signal itself, it is apparent that receivers can be conceived of as manipulating signalers, and in the ‘receiver manipulation’ view, the potential costs to the sender are secondary to the potential benefits to the receiver. A possible benefit for the female túngara frog – the receiver in our example – might be a shorter search time in navigating to the male who adds the more localizable chucks to his calls, perhaps lessening her vulnerability to predation.

**FIGURE 2:**
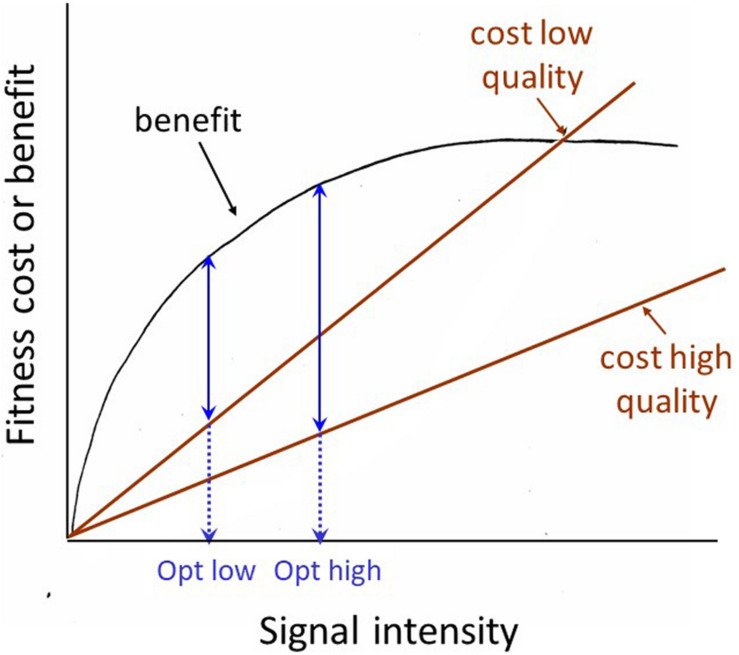
Johnstone’s graphical model of the Handicap principle. The basic assumption is that it costs a high-quality signaler less to signal at its optimum level than it costs a low-quality signaler to signal at that level. The optimum or equilibrium level (where the difference between the costs and benefits of signaling are greatest) for the low quality signaler is lower (opt low) than that for the high-quality signaler (opt high). Thus the signaling level is a reliable indicator of signaler quality.

The receiver manipulation view prompts us to consider how the receiver might demand a more honest signal. There are two related possibilities. First, the receiver can selectively attend to signals that are inherently honest due to physical constraints. For example, in many frogs and toads, size is the most important weapon in male battles over mating opportunities and size is reliably predicted by the pitch of the animal’s vocalization: larger animals give lower-pitched calls. Davies and Halliday (1978) showed that playback of low-pitched calls was sufficient to discourage smaller males from entering into battle with an apparently larger male. A second way to require a more reliable signal has generally been discussed under the rubric of the ‘handicap’ principle. This principle was first proposed by Zahavi (1975), modified and formalized by Grafen (1990), given the intuitively pleasing graphical formulation by Johnstone (1997) shown in Figure 2, and is still being subjected to further modification and clarification (e.g., Penn and Számadó, 2018). But the basic principle is straight-forward, and can be verbalized as follows: signals whose degree of expression is dependent on the health, general condition or vigor of the signaler are inherently honest expressions of that individual’s quality. For a high-quality signaler, a ‘bigger’ signal is a smaller handicap (less costly, or more affordable) than it is for a low-quality signaler, thus ‘big’ signals are reliable signals of signaler quality. One of the clearest demonstrations of honesty in an epigamic signal was carried out by Petrie and her colleagues on that poster animal for epigamic signaling, the peacock. Petrie and colleagues demonstrated that in their peacock population, females preferred a mate with more eyespots in his feather train (whether the difference was natural, or produced by experimental manipulation), and that females mated with males with more eyespots had more young surviving to a year of age than females mated to males with fewer eyespots (Petrie et al., 1991; Petrie, 1994; Petrie and Halliday, 1994). Although the generality of these results has been questioned by studies on other populations (Takahashi et al., 2008; Dakin and Montgomerie, 2011), the example provides a clear illustration of the predictions generated by the handicap principle, and how they should be tested.

The handicap principle should maintain some degree of honesty in any signaling system where signaler and receiver have non-identical interests, such as virtually all mating and agonistic contexts. A low-quality individual can only ‘lie’ by diverting energy into signal development and expression that it needs for maintenance, and so as Searcy and Nowicki (2005) succinctly put it, lying becomes more costly than signaling honestly. Searcy and Nowicki suggest that ‘reliable’ is a better word here than ‘honest,’ for several reasons. First, as with reliability testing in science and elsewhere, we understand that although perfect reliability is unattainable, partial reliability may be good enough. In contrast, ‘honesty’ is generally taken to mean absolute honesty. Second, reliability of a signal is empirically measurable. Thus instead of debating whether an animal signal is informative or not, we can measure if it predicts something important about the present state of affairs or future events. Thus for example, in an agonistic situation a ‘threat signal’ should predict subsequent escalation, and the strongest ‘threat’ signal should predict attack (Searcy and Beecher, 2009).

### Summing Up: Two Perspectives

Historically, the Information Transmission and Manipulation views of animal communication systems have been presented as in opposition. I suggest that in fact they are simply different perspectives on the same process. Once we give the receiver agency, and accept that manipulation is a two-way or reciprocal process in animal communication, we see that the two views have more in common than was at first thought. This rapprochement is nicely captured in the evolution of Dawkins and Krebs’s papers on the topic. In their original paper, Dawkins and Krebs (1978) focused on signalers and argued that “natural selection favors [signalers] who successfully manipulate [receivers] whether or not this is to the advantage of the manipulated individuals.” However, 6 years later in a follow-up paper (Krebs and Dawkins, 1984) they expanded their view to include receiver interests, noting that receivers would be favored to resist manipulation and to attempt to “read the minds” of signalers. Finally, Krebs (1991), discussing Zahavi’s handicap principle, concluded that the manipulation and honest signaling views are probably not incompatible: “Dawkins and Krebs (1978) discussed a coevolutionary process without specifying an end point, whereas Zahavi was concerned mainly with the end-point itself, so it is possible to imagine an evolutionary arms race of manipulation and sales resistance which end up with honest signaling” (Krebs, 1991, p. 67).

**FIGURE 3:**
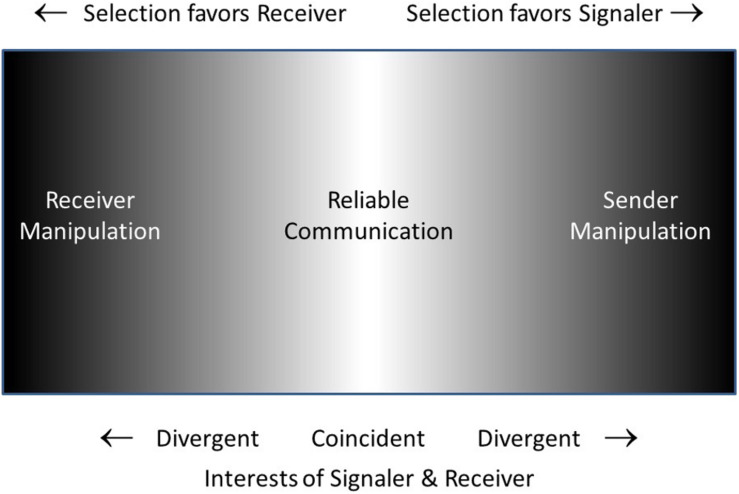
Schematic suggesting the opposing pressures favoring signaler over receiver or vice-versa. Where interests of signaler and receiver are coincident or nearly so (light gray to white) reliable communication will occur. At the extremes of the space (darker), where interests of one or the other of the two parties predominates, signaling will be disfavored. In the intermediate (gray) region, one party may benefit more than the other, but signaling may still be ‘reliable enough.’

Figure 3 is a schematic representation of what I will call the Reciprocal Manipulation view. It shows communication taking place on a battleground in which signaler and receiver are each selected to manipulate the other, the battle being settled in the long run with the compromise of mostly-honest (reliable) signals. The “management-assessment” theory of Owings and Morton (1997, 1998) is quite similar to the Reciprocal Manipulation view. Their theory captures the dynamics of signalers attempting to manage receivers and receivers assessing signalers. In their words “the process of assessment is more active than has been generally recognized, and is responsible for the ‘informational’ couplings between individuals” (1997, p. 359). However, receivers do more than just assess signalers, they manipulate them as well, requiring them to signal in the first place, and requiring a relatively honest signal as a prerequisite for responding to the signal. The Reliable Signaling view of Searcy and Nowicki (2005) is essentially identical to the Reciprocal Manipulation view, with the superficial difference that the former focuses on the information transmission aspect (reliable signaling) while the latter focuses on the manipulation aspect (the conflicting motivations of signaler and receiver).

The Reciprocal Manipulation and Information Transmission views each seem most helpful in different circumstances (Table 3). Where the interests and thus motivations of the two parties differ, the Reciprocal Manipulation highlights the clash. In contrast, where the interests and motivations of the two parties are more in line, the Information Transmission viewpoint focuses on the essence of the interaction. Indeed, where the overlap of sender and receiver interests is considerable, as for example between related individuals, or mates caring for offspring, or individuals in a social group where individuals are strongly interdependent, reliable, mutually beneficial signals will be favored. But even where the interests of sender and receiver are partially opposed, selection acting on both parties will move them to the region where both parties benefit on average, and signals will still be reliable, if less so. This game theory dynamic has been clearly laid out elsewhere (Maynard Smith and Harper, 2003; Godfrey-Smith, 2013).

**TABLE 3:** Differences between reciprocal manipulation and information transmission perspectives.

	Perspective	
	Reciprocal manipulation	Information transmission
Focus on which aspect of the coevolutionary process?	On the process itself	On the end point of the process
Most useful when sender and receiver interests are:	Divergent	Coincident
Focus on what variable?	Differing motivations of sender and receiver	Information transmitted from sender to receiver

I believe that the clash between these views of animal communication has ultimately led us to a clearer view of animal communication systems than the original human-oriented information transmission view. Most animal communication systems are somewhere on the continuum from pure manipulation to pure communication, from arms race (where sender and receiver have different interests, each selected to behave so as to benefit themselves) to pure information transmission (where sender and receiver have identical interests, and where signals benefit or cost both parties in the same way or to the same degree). A fuller development of these ideas can be found in Beecher (2020).

In conclusion, I have argued that we should expect that natural communication systems will generally be reliable, even if not perfectly honest, with signaler and receiver both benefiting on average. However, returning to the main theme of this paper, there is no reason to expect such systems to blossom into simple languages unless signalers and receivers have identical or near-identical interests, and if the ecological selective context requires strong cooperation. There are cognitive prerequisites as well – otherwise one might predict that honeybees should have a simple language – but the brake on the evolution to language-like signaling systems in species with the requisite cognitive capacity is provided by the generally divergent interests of signaler and receiver. Otherwise, bonobos, dolphins and some other vertebrates who seem to have the necessary cognitive prerequisites would have a more language-like natural communication systems than they do.

## Why Are There No Natural Language Systems in Animals?

Research on teaching animals simple human language indicate that at least some animals appear to have the cognitive capacity to decode language or language-like expressions. Herman’s dolphins could comprehend a sign language command such as “take the ball to the hoop” and to distinguish it from a similar but syntactically different command like “take the hoop to the ball” (Herman, 2010). Kanzi the bonobo could respond correctly to novel verbal commands such as “Can you put the pine needles in the refrigerator?” (Savage-Rumbaugh et al., 1993). Pepperberg (1981, 1987) and Pailian et al. (2020) have shown that African gray parrots can follow verbal directions to solve difficult problems, including some that challenge humans. Yet despite having the apparent capacities, at least to some extent, no non-human animal uses even a rudimentary language in its day-to-day existence. This includes groups like the songbirds that seem to have a crucial design feature, the learning and cultural transmission of a complex set of vocal signals. Some animals appear to be smart enough, or capable enough to handle a simple language, but we have yet to discover an animal communication system – in nature – that rises to this level. Thus it appears that some missing element other than cognitive or motor limitations has blocked language evolution in non-human animals. Although it is possible that yet some other cognitive limitation has not been clearly identified (Hauser et al., 2002; Pinker and Jackendoff, 2005), I focus in this final section on a candidate for the missing element that is not purely a cognitive mechanism.

A clue as to the missing element comes from the honeybee ‘dance language.’ Despite a relatively simple nervous system, honeybees are able not only to transmit precise information about events in the external world, but also to use this system in two very different contexts (when talking about the location of desirable food sources or about the location of suitable hive sites). The key ingredient for the evolution of this system, I would argue, is zero conflict of interest between sender and receiver. Both scout and recruit are sister sterile workers and they are both working to feed sisters and brothers slated to be future reproductives. Humans also evolved in a social system featuring extraordinary levels of cooperation, but significantly this cooperation was not restricted to close relatives, as it is in the honeybees and other social insects, ruling out kin selection as a sufficient explanation (but see Fitch, 2004).

I will reframe the question from “why not them?” to the question of “why us” (phrasing suggested by Hrdy, 2009)? How did the human animal become the one species to evolve language? As I argued in the previous section, the field has arrived at a consensus concerning the factors that shape animal communication systems: the pressure for sender and receiver each to shape the interaction to its benefit inevitably both stimulates and constrains the evolution of the communication system. Very unusual circumstances are required for a true language system to evolve. Three essential conditions have to be met. First, the species must have the underlying cognitive capacity. Honeybees may lack this, but some other animals may have it. Second, and this is the clue provided by honeybees, sender and receiver must have identical or near identical interests. Third, individuals must have a compelling need to transmit information across multiple contexts. These are precisely the conditions that existed in pre-human and early human hunter-gatherer societies, the context in which humans and our hominid precursors spent some 95% of our evolutionary history. The description of the prototypical hunter-gatherer society that follows is based on information from a number of sources (including Boehm, 1999; Bowles, 2006; Hrdy, 2009; Hill K. et al., 2011; Knight and Power, 2011; Lee, 2018).

Our hunter-gather ancestors lived in small social groups where individuals were strongly interdependent, and cooperation across multiple contexts was essential for survival. Most highly cooperative animal societies such as the eusocial insects are typically just very large families, but the human hunter-gatherer societies we know – and which we assume to be typical of the ancestral type – consisted of members of several kin lines. Thus human societies then – and now as well – required extensive cooperation among unrelated individuals. Humans are the supreme cooperators in the animal world, but because this cooperation is not supported by high kin relatedness, it has to withstand a strong undercurrent of individual competition. We sometimes lose sight of the human affinity for within-group cooperation because of its paradoxical coexistence with intense between-group competition and tribalism. Irreconcilable conflicts within ancestral hunter-gatherer groups surely occurred, but were often resolved by individuals leaving one group for another (hunter-gatherer societies being classic examples of fission-fusion societies).

Students of human evolution, while differing as to what were the key selective contexts, or the key adaptations, all agree that human evolution has been characterized by remarkable levels of within-group cooperation among unrelated individuals, on a scale not seen in any non-human animal. Several contexts stand out as crucial for the high level of cooperation found in hunter-gather societies. They begin, of course, with hunting and gathering. Effective group hunting (usually done by men) requires sharing of information about distant prey and discussion of strategies for capturing prey. In essentially the same way, gathering of plants and fruits (usually done by women) requires the ability to track the growing schedules and locations of many plants and fruits in the area and the ability to discuss and coordinate foraging activities efficiently. Furthermore, hunter-gatherer societies periodically have to pick up and move to a new, more abundant locale. These moves require discussion and group consensus, with input from all parties, especially older, more experienced men and women.

A second, equally important axis of cooperation is child-raising. Humans are unique among primates in the time and cost required to raise an offspring. Humans solved this problem by involving the whole group in the process. Hrdy (2009) has pointed out that this pattern of cooperative breeding sets humans apart from the exclusive mother-centered parenting of our closest relatives, the great apes. In these early human societies, many individuals played a role in the cooperative care. For starters, the whole group participated in that food brought back to the camp was typically shared among all individuals, without reference to their role in procuring the food. Then unlike most mammals, the father participated in child care alongside the mother. Other relatives were routinely involved in direct child care, especially older siblings and grandparents, often aunts and uncles too, and sometimes non-relatives as well.

Finally, within-group cooperation is essential for success in between-group competition, warfare in particular. This aspect of our hunter-gather heritage is strongly debated in anthropology. Using the terms of Lee (2018), the Peaceful school views significant inter-group competition as not beginning until the Agricultural era, when property gave humans something to fight over. The Bellicose school (e.g., Kelly, 2000; Gat, 2015) believes inter-group competition dates further back in our evolutionary past. But whenever it started, warfare would certainly promote adaptations for within-group cooperation.

In recent years various investigators have proposed key adaptations that may have allowed human societies to achieve this high level of cooperation in the absence of the glue of a very high level of kinship. Although there is not complete agreement as to which of these adaptations were most crucial, taken together they coalesce into a suite of psychological adaptations that promote prosocial within-group interactions within a context of near-complete interdependence. Indeed, Tomasello et al. (2012) have dubbed this the Interdependence hypothesis. The specific adaptations include: shared intentionality (Tomasello et al., 2005), egalitarianism (Boehm, 1999), social learning and communication (Herrmann et al., 2007), intersubjectivity and empathy (Hrdy, 2009), moral intuitions (Haidt, 2012), adaptations for teaching and receiving teaching, and thus cultural transmission (Sterelny, 2012; Henrich, 2016; Whiten, 2017), proactive aggression (Wrangham, 2018) and self-domestication (Wrangham, 2019). These adaptations of our social mind appear to be what set us apart from the other great apes, who it has been argued are otherwise just as cognitively advanced (Herrmann et al., 2007). This suite of adaptations has enabled us to live in complex, cooperative societies. Despite our equally extraordinary proactive (deliberate and planned) aggressive tendencies, directed typically at out-groups, as in wars, pogroms, crusades and the like (Wrangham, 2018), no other social animal has achieved the level of within-group docility and cooperation without high within-group relatedness that is found in the human species. I note that Knight (2018) has an advanced an argument similar to the one I have presented here.

Language unquestionably represents the pinnacle of evolved animal communication systems, and as noted at the beginning of this section, attempts to teach language to animals have not significantly changed this view. Language is often given pride of place in human evolution. In this view the other adaptations mentioned above came only after some form of language was in place. I favor the view of Hrdy (2009), that this may well reverse cause and effect. The evolution of language may have only become possible when the posited unique suite of prosocial, communicative and mind-reading adaptations were in place. The crucial importance of communication in the strongly interdependent social system of early humans would have created this prosocial suite of adaptations, and would have laid the groundwork for evolving a true language.

## Author Contributions

The author confirms being the sole contributor of this work and has approved it for publication.

## Conflict of Interest

The author declares that the research was conducted in the absence of any commercial or financial relationships that could be construed as a potential conflict of interest.
